# Editorial: Long term effects of prenatal exposure to medications and vaccines

**DOI:** 10.3389/fphar.2024.1359539

**Published:** 2024-01-19

**Authors:** Marleen M. H. J. van Gelder, Helga Zoega, Jacqueline M. Cohen

**Affiliations:** ^1^ Department for Health Evidence, Radboud University Medical Center, Nijmegen, Netherlands; ^2^ PharmacoEpidemiology and Drug Safety Research Group, Department of Pharmacy, Faculty of Mathematics and Natural Sciences, University of Oslo, Oslo, Norway; ^3^ School of Population Health, Faculty of Medicine and Health, UNSW Sydney, Sydney, Australia; ^4^ Centre of Public Health Sciences, Faculty of Medicine, University of Iceland, Reykjavík, Iceland; ^5^ Department of Chronic Diseases, Norwegian Institute of Public Health, Oslo, Norway; ^6^ Centre for Fertility and Health, Norwegian Institute of Public Health, Oslo, Norway

**Keywords:** child health, in-utero exposure, medication, neurodevelopment, pharmacoepidemiology, pregnancy, research methods

## 1 Introduction

Although nearly 9 in 10 pregnant individuals use medication (Lupattelli et al., 2014), only 5% of medications have been adequately monitored, tested, and labelled with safety information for use during pregnancy and lactation (Byrne et al., 2020). Most existing evidence is, moreover, focused on immediate birth outcomes following exposure to medication during pregnancy, whereas there are clear indications that some medications may also affect long-term health outcomes in offspring (Kwok et al., 2022; Dreier et al., 2023). This lack of knowledge hampers evidence-based benefit-risk assessment of prescribing and using medications in clinical practice.

Because of ethical and medico-legal concerns regarding medication effects on the fetus, pregnant individuals are routinely excluded from randomized clinical trials (RCTs). In addition, outcomes necessitating long-term follow-up are generally not included or suitable for RCTs. Therefore, we must rely on observational studies to get more insight into the benefits and risks of medication use during pregnancy. Causal inference from observational research designs is indeed challenging, with the potential for confounding, particularly confounding by indication, misclassification of exposures, outcomes, and covariates, and selection bias. The latter may not only refer to selection into the study, but also selective loss-to-follow-up in studies on long-term outcomes.

Within this Research Topic, we aimed at publishing both methodologic and clinical studies on long-term outcomes in offspring after prenatal exposure to medication and vaccines. The Research Topic was published on 28 October 2021, with an extended manuscript deadline on 15 January 2023.

## 2 Outline of contributions

In total, we accepted 6 manuscripts originating from Europe (5 studies) and North America (1 study). Exposures of interest included acetaminophen, opioids, and psychotropic medication; no studies focused on vaccines during pregnancy. Child neurodevelopment was the most commonly assessed outcome (5 studies), whereas childhood asthma was the outcome of interest in one study. Here, we provide an overview of the contributions.

### 2.1 Methodological contributions

As part of the ConcePTION project, Bromley et al. conducted a modified Delphi study among 25 experts to reach consensus on core neurodevelopmental outcomes, optimization of methodology and barriers to conducting pharmacoepidemiologic studies on the safety of medication use with regard to neurodevelopmental outcomes. They provide a set of 11 recommendations, which not only emphasize the need for including neurodevelopmental risk as a safety outcome, but also outline the domains of neurodevelopment that should be considered and provide recommendations for study design features that will improve pregnancy pharmacovigilance on long-term outcomes.

Lupattelli et al. hypothesized maternal personality to be an important confounder in observational studies on long-term effects after prenatal medication exposure. To test this hypothesis, they assessed the relationship between five maternal personality traits and selected medications, showing that the use of antidepressants, benzodiazepines/z-hypnotics, opioid analgesics, and extended use of acetaminophen during pregnancy were more common among women with high neurotic trait. Using the E-value, the strength of these associations helps researchers in estimating the influence of unmeasured confounding when studying long-term effects of medication use during pregnancy.

In a follow-up study among 216 mother-child dyads, Smith-Webb et al. focused on outcome misclassification by assessing whether associations of prenatal acetaminophen exposure with behavioral outcomes were consistent over time and between reporters. They observed that this was not the case: associations were slightly stronger based on parent-reported outcomes during childhood (ages 5–10 years) compared to teacher-reported outcomes. These associations attenuated during adolescence (ages 11–17 years), indicating that also timing of outcome assessment matters. Furthermore, they show that selection bias due to loss-to-follow-up may be important to address.

### 2.2 Safety studies

Using data collected in the Avon Longitudinal Study of Parents and Children (ALSPAC), Golding et al. studied associations between prenatal exposure to acetaminophen and scholastic abilities in children up to 14 years of age. Their results indicate that acetaminophen use in the latter part of pregnancy may be associated with slightly poorer scholastic abilities among girls, particularly in mathematics. However, the effect sizes were very small (around 0.1 standard deviations), with the potential for residual confounding to explain such differences.

Hartwig et al. studied the association between antidepressant use in pregnancy and attention-deficit/hyperactivity disorder (ADHD) in offspring. They employed a case-control sibling design to adjust for confounding by indication, as maternal psychiatric conditions and genetic risk factors are thought to play a role in the etiology of ADHD. In this study, no association between prenatal exposure to antidepressants and ADHD was observed, but the role of the underlying disorder remains to be elucidated.

In a Scandinavian cohort study based on registry data, Odsbu et al. assessed whether prenatal opioid exposure, opioid analgesics or opioid maintenance therapy, were associated with the risk of childhood asthma. The results did not indicate associations between prenatal opioid exposure and childhood asthma, which were generally robust in sensitivity analyses. The authors also showed the importance of choosing relevant comparison groups and applying appropriate statistical approaches to tackle confounding by indication.

## 3 Concluding remarks

The manuscripts included in this Research Topic highlight some of the unique challenges in studying long-term effects of medication use during pregnancy, particularly those related to outcome assessment and confounding. Reassuringly, in most studies no effects of prenatal medication exposure on long-term child health were observed after achieving covariate balance between the comparison groups, emphasising the need for rigorous methods for confounding control. However, the impact of multiple additional potential sources of confounding remain to be explored, such as genetic confounding and the role of the underlying maternal condition. Furthermore, it seems that the influence of selective loss-to-follow-up should not be ignored.

As such, the contributions nicely illustrated the pros and cons of both primary data as well as registry data. Whereas the former may be more complete with respect to confounders specific to effects of prenatal medication exposure on long-term outcomes, sample size and follow-up rates favour registry studies. As many questions with respect to long-term safety of medication and vaccines during pregnancy remain unanswered, we would like to advocate triangulation to overcome the biases arising from a single study design to enable improved benefit-risk assessment in the treatment of women, pregnant people, and individuals planning pregnancy.
